# Early Post-Prandial Regulation of Protein Expression in the Midgut of Chagas Disease Vector *Rhodnius prolixus* Highlights New Potential Targets for Vector Control Strategy

**DOI:** 10.3390/microorganisms9040804

**Published:** 2021-04-11

**Authors:** Radouane Ouali, Larissa Rezende Vieira, Didier Salmon, Sabrina Bousbata

**Affiliations:** 1 Proteomic Plateform, Laboratory of Microbiology, Department of Molecular Biology, Université Libre de Bruxelles, 6041 Gosselies, Belgium; 2 Laboratory of Molecular Biology of Trypanosomatids, Institute of Medical Biochemistry Leopoldo de Meis, Centro de Ciências da Saúde, Federal University of Rio de Janeiro, Rio de Janeiro RJ 21941-902, Brazil; larissanat@hotmail.com (L.R.V.); salmon@bioqmed.ufrj.br (D.S.)

**Keywords:** chagas disease, *Rhodnius prolixus*, hematophagy, label-free shotgun, vector control

## Abstract

Chagas disease is a vector-borne parasitic disease caused by the flagellated protozoan *Trypanosoma cruzi* and transmitted to humans by a large group of bloodsucking triatomine bugs. Triatomine insects, such as *Rhodnius prolixus*, ingest a huge amount of blood in a single meal. Their midgut represents an important interface for triatomine–trypanosome interactions. Furthermore, the development of parasites and their vectorial transmission are closely linked to the blood feeding and digestion; thus, an understanding of their physiology is essential for the development of new strategies to control triatomines. In this study, we used label-free quantitative proteomics to identify and analyze the early effect of blood feeding on protein expression in the midgut of *Rhodnius prolixus*. We both identified and quantified 124 proteins in the anterior midgut (AM) and 40 in the posterior midgut (PM), which vary significantly 6 h after feeding. The detailed analysis of these proteins revealed their predominant involvement in the primary function of hematophagy, including proteases, proteases inhibitors, amino acids metabolism, primary metabolites processing, and protein folding. Interestingly, our proteomics data show a potential role of the AM in protein digestion. Moreover, proteins related to detoxification processes and innate immunity, which are largely accepted to be triggered by blood ingestion, were mildly modulated. Surprisingly, one third of blood-regulated proteins in the AM have unknown function. This work contributes to the improvement of knowledge on the digestive physiology of triatomines in the early hours post-feeding. It provides key information for selecting new putative targets for the development of triatomine control tools and their potential role in the vector competence, which could be applied to other vector species.

## 1. Introduction

Hematophagous arthropods constitute the most relevant veterinary, sanitary, and epidemiologically insect group related to vector-borne diseases [1]. They can transmit a wide range of pathogenic agents which cause different types of diseases and infections, affecting livestock, domestic animals, and people [2]. Chagas disease is the most important parasitic infection in Latin America and it is ranked as one of the most important neglected tropical disease worldwide [3]. In fact, it affects about 8 million people and causing more than 14,000 deaths annually (World Health Organization, 2020) [4]. Moreover, it is becoming an emerging public health problem in non-endemic areas due to the intensification of population movement [5]. 

The causative agent of this disease is the flagellate protozoan parasite *Trypanosoma cruzi*, which is mainly transmitted to humans by hematophagous triatomine bugs [6]. *Rhodnius prolixus* is an important model for triatomine physiology and for the study of the transmission of Chagas disease [7]. Because no vaccine is available, prophylactic methods such as vector control represent the most efficient way to reduce the incidence of the disease. However, triatomine resistance to insecticides is a complex problem involving different resistance mechanisms [8,9,10]. For these reasons, the development of new tools and methods to block the transmission of Chagas disease is primordial. 

The digestive system of triatomines is the organ responsible of host’s blood digestion and nutrients absorption [11,12]. It is also the site for *T. cruzi* development, being thus an important target for parasite transmission control [13]. The digestive tract comprises three distinct compartments: anterior midgut (AM), posterior midgut (PM), and rectum (RE). The AM is the first segment of the digestive tract where blood is stored and erythrocytes are lyzed [3,11,14]. In addition, clotting of the ingested blood is inhibited by intestinal anticoagulant molecules expressed in the AM [15,16,17,18]. Recent studies on *R. prolixus* AM have also shown the presence of a proteolytic activity in this tissue [19,20]. Triatomines AM also contains most of the bacterial microbiota, the number of which increases dramatically after a blood meal [11,13,21]. The PM is the digestive and absorptive organ of the midgut [11]. It has also an important role in the development of *T. cruzi*. In this region, the epimastigote forms multiply and attach to the perimicrovillar membrane (PMM) which covers the intestinal cells [3,13,22,23]. Finally, the RE that ensures excretion processes is also the place where the highest population densities and the cellular differentiation of the epimastigotes into metacyclic trypomastigotes (metacyclogenesis) occurs [22,24,25]. 

Blood feeding induces several changes in triatomine’s gut environment, such as changes in temperature, osmolarity, pH, and oxidative stress [6,14]. All these changes could make this organ a hostile environment for the development of *T. cruzi*. Indeed, a significant microbiota-independent mortality of *T. cruzi* was observed in the AM during the first 6 h after feeding [23,24]. Therefore, it has been hypothesized that molecules expressed in triatomine AM after blood meal ingestion could be responsible directly or indirectly for parasitic killing [23,24].

Despite numerous studies on triatomines, many questions about their digestive physiology and interaction with *T. cruzi* remain unanswered. In this context, the study of the effect of blood ingestion on changes in protein expression in the midgut of *R. prolixus* is expected to provide new insights into the key cellular and molecular processes of the digestive physiology which are triggered in the early hours following the arrival of blood. This should provide valuable information on potential candidates that might be at the origin of new strategies for triatomines control or could affect their vectorial competence for the transmission of *T. cruzi*. To achieve this goal, a differential proteomic study of the midgut tissues of *R. prolixus* from starved and blood fed insects was carried out using a label-free high throughput proteomic approach. Proteins differentially expressed between these two conditions were analyzed, their involvement in the digestive process, and their potential involvement in the interaction with *T. cruzi* parasite are discussed. 

## 2. Materials and Methods

### 2.1. Samples Preparation

*R. prolixus* insects are reared from egg to adults in plastic boxes (26.5 × 15.5 × 8.5 cm^3^) with a voile-covered opening on the lid, at a stable temperature of 28 °C and 60–80% humidity, under a photoperiod of 12 h light/12 h dark. The total development process takes place in about 6 months. Upon reaching adulthood, the insects are transferred to larger boxes (18 × 17 × 19.5 cm^3^) with an average of 200 animals per box at a female/male ratio of 4:1. Adult insects are fed at intervals of 3 weeks with rabbit blood. 

### 2.2. Dissection of the Insect and Tissues Preparation

For the starved insects, the digestive tract was taken 3 weeks after the first meal and for the blood fed insects the digestive tract was dissected 6 h after in vitro feeding with heparinized rabbit blood (2.5 units/mL). Three biological replicates per condition, each of five randomly selected females, were used. Insects were dissected in cold phosphate buffered saline solution (0.15 M NaCl, 43 mM Na_2_HPO_4_, 1.4 mM KH_2_PO_4_, 2.7 mM KCl, pH 7.4) and the midgut, carefully pulled apart, was separated in AM (cut after cardia) and PM compartments. AM and PM were incised and rigorously washed with cold phosphate buffered saline to remove intestinal content. Intact midguts were stored in STE buffer (0.1 M Tris, 0.05 M NaCl, 0.05 M EDTA, pH 7.4) at −80 °C until use. 

### 2.3. Proteins Extraction

Freezing/thawing at −80 °C in the storage buffer was sufficient to recover AM and PM soluble proteins in the supernatant by centrifugation of the cellular debris at 13,000× *g* for 15 min at 4 °C. Protein concentration was determined with Pierce 660 nm Protein Assay (Thermo Scientific Inc. Rockford, IL, USA) using a series of bovine serum albumin (BSA) solutions as protein concentration standards. Starved and blood fed biological replicates were processed separately throughout the whole analyzes.

### 2.4. Sample Preparation Prior to Liquid Chromatography Tandem Mass Spectrometry

Ten micrograms of proteins from each biological replicate of each nutritional condition were mixed with lysis buffer (20 mM HEPES, 8 M urea, 0.5 M dithiothreitol, pH 8.0), the solution was mixed, and incubated at 55 °C for 30 min. Then, 0.5 M of iodoacetamide were added and the sample was incubated for 15 min at room temperature in the dark. The samples were diluted 2-folds with 20 mM HEPES, pH 8.0 prior to enzymatic digestion. The samples were digested first by 1 µg of LysC (Promega, Leiden, The Netherlands) for 4 h at 37 °C, then by 1 µg of trypsin (Promega, Leiden, The Netherlands) overnight at 37 °C. Digestion was stopped by adding 1% trifluoroacetic acid (TFA). The resulting peptide mixture was purified using OMIX C_18_ pipette tips (Agilent, Santa Clara, CA, USA). The purified peptides were dried completely and re-suspended in 20 µL loading solvent A (0.1% TFA in water/acetonitrile, 2/98 (*v*/*v*)) of which 10 µL were injected for LC-MS/MS analysis on an Ultimate 3000 RSLC nano-ProFlow system on-line connected to a Q Exactive HF mass spectrometer (Thermo Fisher, Waltham, MA, USA). Trapping was performed at 10 μL/min. for 4 min in the loading solvent A on a 2 cm trapping column (made in-house, 100 μm internal diameter, 5 μm beads, C_18_ Reprosil-HD, Dr. Maisch, Germany). The peptides were separated on a 20 cm µPAC™ column with C_18_-endcapped functionality (Pharmafluidics, Gent, Belgium) kept at a constant temperature of 50 °C. Peptides were eluted by a non-linear gradient at 9% solvent B (0.1% formic acid in water/acetonitrile (2/8, (*v*/*v*)) for 15 min, 30% solvent B for 105 min., 55% solvent B for 125 min and 99% solvent B for 135 min at a constant flow rate of 300 nL/min, followed by a 5 min wash at 99% solvent B. The mass spectrometer was operated in data-dependent mode, automatically switching between MS and MS/MS acquisition for the 16 most abundant ion peaks per MS spectrum. Full-scan MS spectra (375–1500 m/z) were acquired at a resolution of 60,000 in the Orbitrap analyzer after accumulation to a target value of 3.10^6^. The 16 most intense ions above a threshold value of 13,000 were isolated with a width of 1.5 m/z for fragmentation at a normalized collision energy of 28% after filling the trap at a target value of 100,000 for maximum 80 ms. MS/MS spectra (200–2000 m/z) were acquired at a resolution of 15,000 in the Orbitrap analyzer.

### 2.5. Mass Spectrometric Data Analysis

Protein identification from the MS data was realized with the Andromeda peptide database search engine integrated into the computational proteomics platform MaxQuant (version 1.6.3.4, Max Planck Institute of Biochemistry, Planegg, Germany) [26] with default search settings including a false discovery rate set at 1% on both the peptide and the protein level. Spectra were searched against *R. prolixus* (UniProt Tax ID: 13249) proteins in the UniProt/Swiss-Prot reference database (UniProt Proteome ID: UP000015103) and the decoy database. Andromeda search parameters for protein identification specified a first search mass tolerance of 20 ppm and a main search tolerance of 4.5 ppm for the parental peptide. Enzyme specificity was set to C-terminal to arginine and lysine, also allowing cleavage at arginine/lysine-proline bonds with a maximum of two missed-cleavages. Variable modifications were set to oxidation of methionine and acetylation of protein N-termini. A minimum of one unique peptide was required for identification. We allowed for matching between runs using a 1.5 min match time window and a 20 min alignment time window. Proteins were quantified by the MaxLFQ algorithm integrated in the MaxQuant software. A minimum ratio count of two unique or razor peptides was required for quantification.

Further data analysis was performed with the Perseus software (version 1.6.2.1, Max Planck Institute of Biochemistry, Planegg, Germany) after loading the protein groups file obtained previously by MaxQuant software. First, proteins identified by site and reverse database hits were removed and label-free quantitation (LFQ) values were log2 transformed to achieve normal data distribution. Data from three biological replicates of both starved and 6 h post-fed conditions were grouped as two different conditions (starved and blood fed) and proteins with less than 3 valid values in at least one condition were removed. Then, missing values from the other condition were imputed with values from the lower part of the normal distribution representing the detection limit. Statistical significance of changes in abundance between sample groups was calculated by a two-tailed t-test, with *p*-values adjusted for multiple testing by a permutation-based FDR at 5%. Microsoft Excel was used to calculate ratios and fold-changes (FC) followed by log2 transformation. Only proteins with FC ≥ 2 and *p*-value ≤ 0.05 were considered. Results are visualized by Volcano plots. A list of differentially expressed proteins generated by Perseus software containing proteomic identification parameters (unique peptides, sequence coverage percentage, identification score and calculated FC) was then created.

### 2.6. Functional Characterization and Protein Classification

UniProt ID numbers from the protein list generated by Perseus was searched against UniProtKB using Retrieve/ID mapping tool (https://www.uniprot.org/uploadlists, 4 November 2020). This allows to associate UniProt accession the corresponding protein names, gene ontology categories and their IDs, molecular functions, biological processes, and VectorBase IDs. Protein classification was then performed according to Gene Ontology (GO) hierarchy, using PANTHER (Protein ANalysis THrough Evolutionary Relationships) classification system (http://www.pantherdb.org/, 6 November 2020) [27]. 

### 2.7. Western Blotting

Fifteen μg of *R. prolixus* AM and PM proteins were labeled with 0.5 μg of CY5 (Lumiprobe, Hannover, Germany) for 30 min. in the dark (four replicates for each condition were used). The reaction was stopped with 0.5 μg of lysine and the proteins were denatured in Laemmli buffer (0.125 M Tris HCl, 4% SDS, 20% glycerol, 10% 2-mercaptoethanol, 0.004% bromphenol blue). Then they were deposited on 10% SDS-PAGE gel. After separation, the proteins were transferred into a nitrocellulose membrane (Thermofisher, Merelbeke, Belgium) by electro-transfer for 75 min. at 150 V at 4 °C, using the transfer buffer (0.25 M Tris, 200 mM Glycine, 20% methanol). CY5 labeling was revealed at 600 nm by digital imaging with a CCD camera (Odyssey^®^ Fc, Bad Homburg, Germany) to quantify the total proteins. The membrane is then saturated overnight with TBS (20 mM Tris, 150 mM NaCl, pH 7.6) in the presence of 5% milk and 0.1% Tween20. The membrane was incubated for 3 h with the appropriate primary antibodies in a solution of TBS, 2.5% milk and 0.05% Tween20. The membrane was then washed 5 times with TBS, 2.5% milk and 0.05% Tween20 to remove excess antibodies before introducing the secondary antibody conjugated to horseradish peroxidase. The membrane was washed 3 times with a solution of TBS, 2.5% milk and 0.05% Tween20 and twice with TBS. The bound antibodies were detected by chemiluminescence. The light produced by the enzymatic reaction is detected by digital imaging with a CCD camera (Odyssey^®^ Fc, Bad Homburg, Germany). The relative expression of target proteins was calculated by comparing the signal strength of the target protein to the total protein signal measured by either CY5 or Ponceau S reading. The significance of the expression difference between the two conditions was calculated based on the difference of the means values between the two conditions using Student’s *t*-test. The results were analyzed using GraphPad Prism Software, version 5.00. Differences between starved and blood fed groups were considered statistically significant when *p* ≤ 0.05. Semi-quantitative Western blot analyses were based on four independent biological replicates.

## 3. Results

### 3.1. Differential Protein Expression between Starved and Blood Fed Conditions

**Figure 1:**
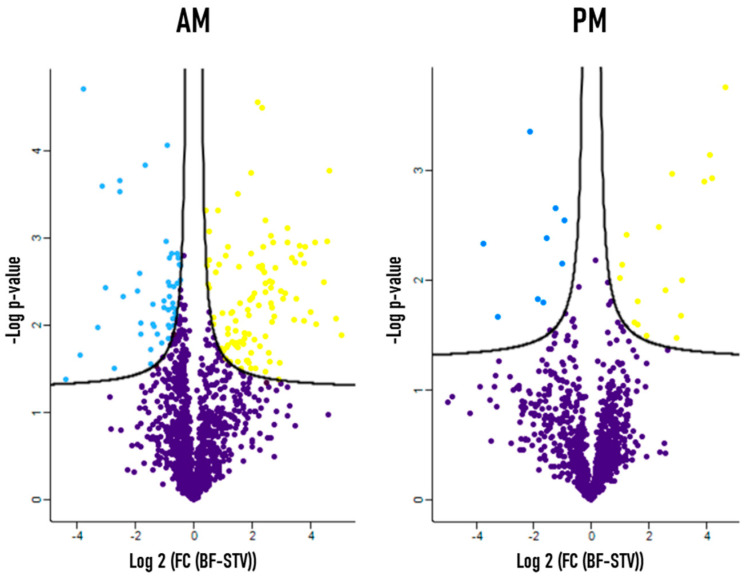
Volcano plot comparing the normalized expression of proteins in *R. prolixus* anterior midgut (AM) (left) and posterior midgut (PM) (right) between starved and 6 h post blood feeding conditions. *y*-axis: negative log of *p*-value; *x*-axis: log2-transformed fold-change; yellow dots: up-regulated proteins with significant *p*-value; blue dots: down-regulated proteins with significant *p*-value; purple dots below the significance lines: non-variable proteins. Differentially expressed proteins were determined by Student’s *t*-test (*p* ≤ 0.05) and FC ≥ 2.

One of the main objectives of this study is to better understand the digestive physiology of *R. prolixus* by analyzing on a large scale the early effect of the blood meal on protein expression in the midgut. To achieve this goal, a differential proteomic study of protein expression comparing starved and 6 h post-fed *R. prolixus* midgut tissues by label-free shotgun proteomics approach was carried out. This analysis allowed the identification of 124 proteins to be significantly differentially expressed in the AM, among which 100 were up-regulated and 24 were down-regulated after a meal ingestion. Similarly, 40 blood-regulated proteins were identified in the PM, 25 of which were up-regulated while 15 of which were down-regulated after blood feeding. All these proteins were identified with at least one unique peptide, and their expression level showed at least two-fold change (FC) in one of the two compared physiological condition (FC ≥ 2). A volcano plot displaying this variation is shown in Figure 1 and the corresponding list of proteins, whose level of expression is regulated by the blood meal, is presented in Table S1. The proteomic identification parameters of each protein, including the number of unique peptides, which allowed the identification, the percentage of sequence coverage and the identification score are presented. The fold change in protein expression level between starved and blood-fed conditions was calculated by measuring the difference in LFQ intensities between the two conditions (see Material and Methods) and was ranging between 2 and 32-folds (Table S1).

### 3.2. Functional Annotation of Blood Regulated Proteins

**Figure 2:**
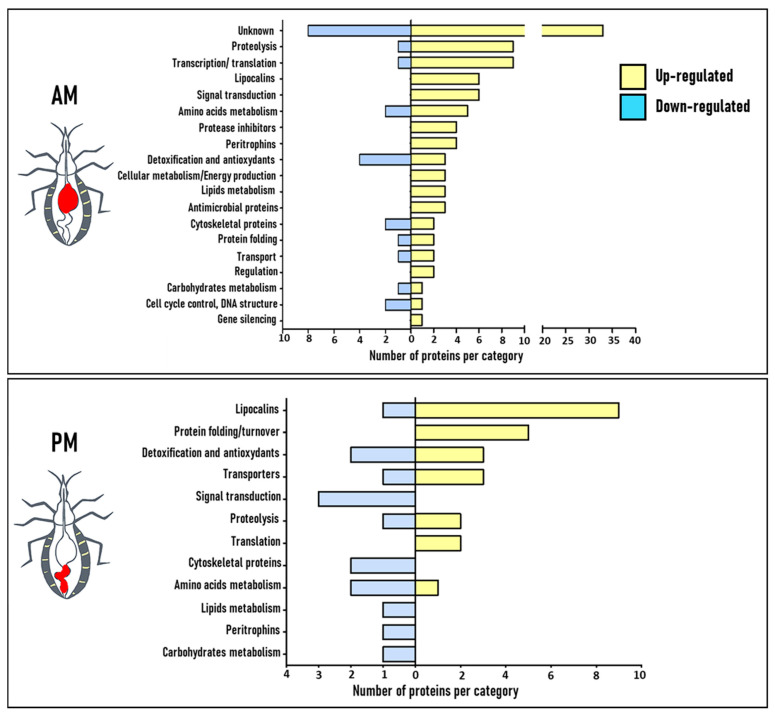
Functional annotation of differentially expressed proteins between starved and blood fed conditions in *R. prolixus* AM (upper panel) and PM (lower panel) tissues. The proteins have been classified according to their physiological and/or cellular function according to Gene Ontology and the literature. Table S1 provides details on Gene Ontology (GO) analysis and classification.

Differentially expressed proteins were classified into functional groups according to their physiological function based on information obtained from Gene Ontology classification (GO, http://geneontology.org, 22 November 2020) (Figure 2). In addition, new functional categories of proteins have also been created based on the literature (Table S1). 

**Figure 3:**
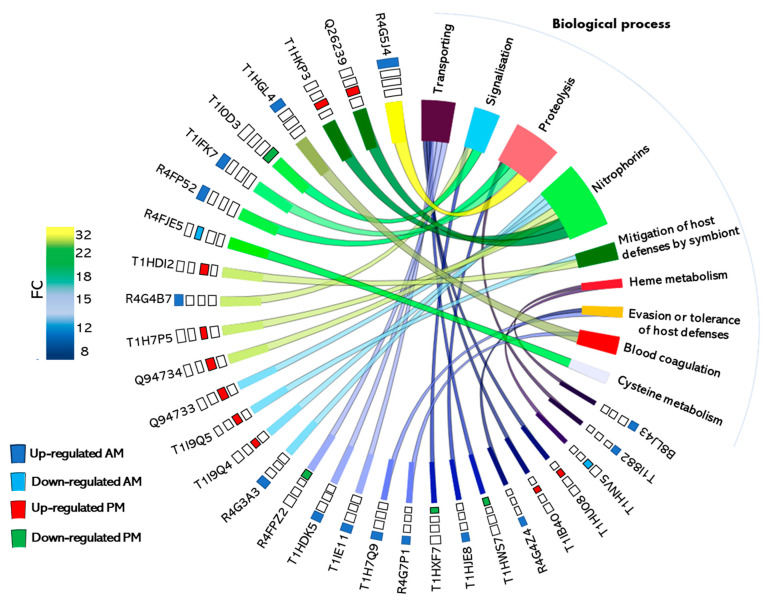
Chord diagram presenting top ranked differentially regulated proteins in *R. prolixus* midgut tissues in response to blood feeding. Proteins are linked to their assigned biological process via colored ribbons. Proteins are ordered according to the calculated fold-change (FC), which is displayed in descending color intensity with a cut-off fold-change ≥ 8.

Surprisingly, in the AM, about one third of proteins whose expression is regulated by blood ingestion were of unknown function (Figure 2, upper panel). Indeed, out of the 124 differentially expressed proteins, 41 were classified in this category due to the absence of GO and domain function (Table S1). A total of 33 proteins of them were up-regulated after the blood meal and 8 were down-regulated. The remaining proteins belong to a wide range of functional classes with among them, the proteases and proteins involved in gene expression (transcription/translation), which are the most represented among the up-regulated functional categories, each representing 9% of the up-regulated proteins after blood ingestion. They are followed by lipocalins and proteins involved in signal transduction mechanisms (each representing 6% of the up-regulated proteins), proteins involved in amino acids metabolism (5% of the up-regulated proteins), proteases inhibitors, and peritrophins (each representing 4% of the up-regulated proteins). The other minor functional categories are represented by proteins involved in detoxification processes, in processing of primary metabolites (carbohydrates and lipids), cellular metabolism, protein folding, antimicrobial response, transporters, cytoskeletal modulation, regulation, cell cycle control, and gene silencing. The relative level of the variation in expression of these proteins in response to blood meal ingestion is not uniform. Indeed, the expression of some proteins increases considerably after a blood meal to reach 32-folds compared to the starved condition (Figure 3 and Table S1). However, the expression variation of other proteins was only two-folds (the minimal fold cut-off) in response to the blood meal. The protein categories with the most important fold-changes are proteases (3 ≤ FC ≤ 32), proteins with unknown function (2 ≤ FC ≤ 29), protease inhibitors (4 ≤ FC ≤ 24), signal transduction (4 ≤ FC ≤ 18), lipocalins (3 ≤ FC ≤ 15), and lipids metabolism (2 ≤ FC ≤ 13). Proteins whose expression is down-regulated in response to blood ingestion are of unknown function (32%), followed by proteins involved in reactive oxygen species (ROS) detoxification (16%), cell cycle control, amino acid metabolism, and cytoskeletal proteins (each representing 8%). The remaining categories are represented by a single protein, such as proteases and proteins involved in transcription/translation, protein folding, transport, and carbohydrates metabolism.

In the PM, out of a total of 25 blood up-regulated proteins, 36% are lipocalins, 20% are involved in protein folding, 12% in detoxification processes and transport, 8% in proteolysis and translation, and 4% in amino acids metabolism. Concerning the down-regulated proteins, 20% are involved in signal transduction, 13% in detoxification processes, amino acids metabolism, and cytoskeletal modulation. Lipocalins, transporters, proteases, peritrophins, proteins involved in carbohydrates, and lipids metabolism are represented by a single protein. The variation in protein expression in response to blood meal ingestion is ranging from 2 to 26-folds for the up-regulated proteins and 2 to 23-folds for the down-regulated proteins (Figure 2 and Figure 3). Proteins showing the highest variation are lipocalins (8 ≤ FC ≤ 26) and transporters (3 ≤ FC ≤ 9). For the down-regulated proteins, the most important fold-change was observed with proteins implicated in signal transduction (3 ≤ FC ≤ 23), transport (FC = 13), cytoskeleton (7 ≤ FC ≤ 9), and for peritrophins (FC = 9). 

### 3.3. Validation by Western Blot of Differentially Expressed Proteins Candidates 

**Figure 4:**
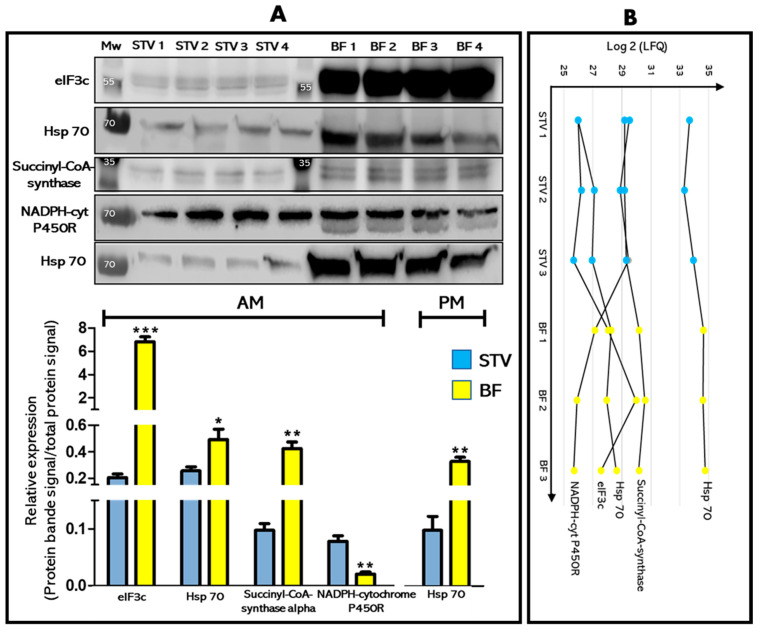
Western blot validation of protein candidates identified as being significantly differentially expressed in the midgut between starved (STV) and blood fed (BF) insects. (**A**) upper panel: Western blot analysis of eIF3c, hsp70, succinyl-CoA-synthase, and NADPH-cytochrome P450 reductase, identified in the AM and hsp70 identified in the PM comparing STV and BF insects. Molecular weight marker is indicated in kDa. (**A**) lower panel: The relative expression of the protein candidates was calculated by normalizing the band intensity of the target protein to the intensity of the total proteins signal. Histogram represents the semi-quantitative Western blot quantification. The results are expressed as the mean ± SEM (*n* = 4), and statistical significance is shown by * *p* ≤ 0.05, ** *p* ≤ 0.01 and *** *p* ≤ 0.001. (**B**) Profile plot representing the Log2 LFQ intensity profiles for the chosen protein candidates comparing the STV and BF conditions (*n* = 3).

Five conserved proteins showing differential expression after a blood meal (STV versus BF) and for which antibodies raised against the corresponding orthologue protein were commercially available have been validated by semi-quantitative Western blot (Figure 4). Three proteins eIF3c (T1I998), hsp70 (T1HJT8), and succinyl-CoA-synthase (R4G324) from the AM and an hsp70 (T1HJT8) from the PM, which expression level is induced by the blood meal according to the quantitative proteomic analysis (Figure 4B) showed that both the expression profile and the magnitude of change by semi-quantitative Western blot is the same (Figure 4A). Similarly, NADPH-cytochrome P450 reductase (T1HNV5) whose expression was down-regulated by the blood meal in the AM was concordant between quantitative proteomic (Figure 4B) and semi-quantitative Western blot (Figure 4A). 

## 4. Discussion

This work constitutes the first in-depth analysis of the effect of a blood intake on protein expression in the midgut of *R. prolixus* 6 h after feeding. This sampling time was selected to monitor the early events occurring in the midgut after a blood meal as a massive lysis of *T. cruzi* was observed in the AM during the first hours post-feeding [23,24]. The midgut is not only the organ of blood digestion, it is also the first site of *T. cruzi* interaction and development in the insect. That property makes it a particularly promising target for the development of vector control strategies. Considering the above, knowledge on the midgut proteome and modulation of protein expression upon feeding and digestion will provide key information for identifying and selecting novel targets for the development of triatomines control tools. For these reasons, proteins which expression is regulated by blood feeding were analyzed and their involvement in digestion and potentially on *T. cruzi* interaction were discussed. 

### 4.1. A Large Blood Meal Has Limited Effect on the Dynamic of Protein Expression in R. prolixus Midgut during the First Hours after Feeding 

In our previous work on the analysis of the midgut proteome of *R. prolixus* using high throughput proteomic tools, we identified 1471 proteins in the AM and 1132 proteins in the PM [20]. There are only 124 proteins showing variation in response to blood meal ingestion in the AM and 40 proteins in the PM (Figure 1) at 6 h post-feeding. These differentially expressed proteins correspond to 8% and 3% of the total proteins expressed in the AM and the PM, respectively. This observation suggests that the proteome of *R. prolixus* remains relatively stable and does not undergo significant variation during the first hours after blood ingestion. The overwhelming majority of proteins are therefore constitutively expressed in these tissues. Despite the absence of large scale studies on the effect of blood on the dynamics of gene expression in triatomines, this observation is in agreement with published comparative proteomics and post-genomics studies in several hematophagous arthropods, such as insects: *Anopheles albimanus* [28], *Anopheles gambiae* [29,30], *Aedes albopictus* [31], *Aedes aegypti* [32], *Phlebotomus perniciosus* [33], and arachnids: *Ixodes ricinus* [34,35], *Ornithodoros erraticus* [36], and *Ornithodoros maubata* [37]. 

### 4.2. Detailed Analysis of the Differentially Expressed Proteins and Their Physiological Involvement in Blood Digestion

To better characterize the early response of *R. prolixus* midgut to the ingestion of an important amount of blood, proteins with important role in blood processing will be discussed. This will provide new insights into the physiology of triatomines and candidates that could be exploited for vector control approach will be suggested. 

#### 4.2.1. The Vast Majority of the Differentially Expressed Proteins in the Midgut in Response to Blood Intake Are Involved in a Physiological Role Related to Blood Processing

- Proteases

Triatomines require proteases as the main enzymes in the midgut lumen for a blood meal processing. This is carried out mainly by cathepsins, especially cysteine proteases (cathepsins B and L), and aspartic proteases (cathepsin D) [11,38]. Cathepsins D are the dominant proteases in the gut in terms of the number of isoforms [20] and expression level upon blood ingestion by *R. prolixus* (Figure 2 and Figure 3). According to bioinformatic analysis [39], the genome of *R. prolixus* encodes 19 genes belonging to the A1 peptidase family [39]. Ten have their expression confirmed at the protein level [20]. Interestingly, seven cathepsins D were up-regulated in the AM (R4G5J4, T1IFK7, R4FP52, T1HJE8, T1I882, T1HY69, and T1I865) while one was down-regulated (T1I913) in response to blood ingestion (Table S1). Their expression level increased drastically after feeding, particularly that of R4G5J4 (32-folds), T1IFK7 and R4FP52 (22-folds), T1HJE8 (9-folds), and T1I882 (8-folds) (Figure 3). Interestingly, R4G5J4, T1IFK7, and R4FP52 with the highest fold increase are branched apart from the other cathepsins D in the phylogenetic trees published by Ribeiro et al. [40] and Henriques et al. [39]. The identification of aspartyl proteases activity in the PM of *R. prolixus* has been associated solely with blood digestion [41]. However, these enzymes may fulfill intracellular roles, such as the hydrolysis of intracellular proteins into lysosomes [40] or perform other functions related to vector fitness. Indeed, cathepsin D gene expression has been observed to increase in *Triatoma infestans* midgut during *T. cruzi* infection [42,43]. Nevertheless, although these proteins were recovered from tissue homogenates, their induction in the AM by blood ingestion suggests a potential digestive role. Indeed, a proteolytic activity has been demonstrated in the AM [19,20]. Curiously, T1I913 (Table S1) was down-regulated upon blood digestion while it resulted in the highest number of transcripts in the AM transcriptome [40]. Although post-transcriptional and translational regulations cannot be excluded, it is reasonable to assume that expression pattern of cathepsin D isoforms is triggered differentially by the sampling time. It is noteworthy to mention that the three invariable cathepsins D (R4FKP9, R4G4V2, and T1HRT9) identified by Ouali et al. [20] are grouped in different clades containing non-hematophagous insects and can therefore play non-digestive functions. We identified one aspartyl protease (T1I914) in the PM, whose expression increases by five-folds after blood feeding. Analysis of the sequence of this protease displays a 75% similarity with TiCatD, an aspartyl protease characterized from the PM of *T. infestans*. The temporal expression pattern of TiCatD in the PM after feeding revealed a feeding dependent regulation [44]. A single cathepsin L-like cysteine protease (R4FM70) was four-folds up-regulated in the PM after feeding (Table S1). This protein shares respectively 82% and 80% similarity with TBCATL-1 and TBCATL-2 from *Triatoma brasiliensis* [45] and 69% similarity with *T. infestans* CatL1 [46].

Serine carboxypeptidase (R4G841) belonging to the peptidase S10 family is three-folds overexpressed in the AM after blood ingestion (Table S1). Although the intracellular digestion of proteins in triatomines is ensured by lysosomal enzymes [38], the overexpression of this protein after blood ingestion suggests its potential implication in the extracellular digestive process. Serine carboxypeptidases are scarcely described in triatomines. However, two serine carboxypeptidases have been described in *T. brasiliensis* midgut and their activity against specific substrate was shown in both AM and PM tissues [47]. Interestingly, their activity is induced after blood meal ingestion only in the PM [47]. This suggests that serine carboxypeptidases contribute to the carboxypeptidase activity present in the midgut lumen of triatomines. However, their physiological implication remains to be confirmed in the AM. 

A metalloendopeptidase (T1HRH4) of the M3 family showed three-folds induction following blood ingestion in the AM (Table S1). There are no studies on the expression profile or the role of metalloproteases in the midgut of triatomines. However, they are known to be involved in extracellular matrix degradation, protein shedding from the cell surface and enzyme activation. Hence, tick salivary metalloproteases accelerate fibrinolysis [48] and inhibit neutrophil function [49]. Moreover, RNAi silencing of metalloproteases in *Rhipicephalus microplus* affects average egg weight and oviposition rate [50]. Two metalloproteases are expressed in the hemolymph of *R. prolixus* infected with *Enterobacter cloacae* [51] and *Trypanosoma rangeli* [52]. These proteases are expressed in the fat body and their release into the hemolymph upon infection suggests their role in *R. prolixus* defense against pathogens [53]. Although the function of these proteins in the midgut is unknown, a function similar to that of the saliva could be expected [48].

- Protease Inhibitors

Protease inhibitors are ubiquitous and play an essential role in the regulation of many biological processes [54]. Their main function in hematophagous species is the regulation of blood coagulation. Indeed, specific anticoagulant molecules such as inhibitors of the coagulation cascade and platelet aggregation are produced in the saliva and the midgut [55]. In triatomines, several protease inhibitors were identified in both the saliva and the digestive tract such as PmStKaz from *Panstrongylus megistus* AM [56], triabin from *Triatoma pallidipennis* saliva [57], infestin and brasiliensin from *T. infestans* [17] and *T. brasiliensis* [16] midguts, respectively. Eleven protease inhibitors with a conserved kazal domain are transcribed in *R. prolixus* digestive tract, and some of them have multiple kazal domains [40]. Several protease inhibitors have been characterized such as rhodniin, which is an efficient thrombin inhibitor through two kazal-type binding domains [15,18]. In this comparative study, we show that rhodniin is a blood regulated protein and its expression increases by five-folds after feeding (Table S1). In addition, two other kazal-type serine protease inhibitorsR4G7P1 with a single kazal domain (10-folds) and T1IGD5 with two kazal domains (four-folds) are induced by the blood meal (Table S1). These protease inhibitors are probably overexpressed in the AM in response to the massive blood arrival to maintain blood fluidity in the insect digestive tract by blocking the coagulation cascade [15]. However, these inhibitors may have another function in the AM, such as interaction with pathogens in the ingested blood. Indeed, an overexpression of *R. prolixus* trypsin inhibitor RpTI has been observed in the AM following its infection with *T. cruzi* [58]. 

Triabin-like lipocalin 4a precursor (T1H7Q9), another inhibitor of thrombin, belonging to lipocalins family increases by 10-folds in the AM after blood ingestion. Triabins are a highly potent exosite inhibitors of thrombin, originally isolated from the salivary gland of the blood sucking bug *T. pallidipennis* [57]. Apart from its thrombin inhibitory function, this protein has been classified in lipocalins family based on structural similarities [59]. The expression of all these anticoagulant proteins makes the AM of *R. prolixus* a source of potential novel functional proteins with pharmacological interest.

- Amino Acids Metabolism

As the blood meal is mainly constituted by proteins, its digestion results in the release of important quantities of amino acids. Thus, amino acid metabolism is remarkably important in hematophagous arthropods because of their protein-rich diet. This particular feeding behavior offers an abundance of nutrients but the hydrolysis of blood proteins generates as well toxic concentrations of heme and amino acids, such as tyrosine [60]. Interestingly, tyrosine aminotransferase (TAT) (R4G400), the first key enzyme in the tyrosine degradation pathway, is up-regulated by three-folds after blood feeding in the AM (Table S1). A recent study demonstrated that tyrosine detoxification is an essential trait for survival in blood sucking arthropods [61]. Indeed, gene silencing of *R. prolixus* TAT and 4-hydroxyphenylpyruvate dioxygenase (HPPD), the first two enzymes in the phenylalanine/tyrosine degradation pathway, caused the death of insects after a blood meal [61,62]. Given the major importance of tyrosine metabolism for the digestive physiology of hematophagous insects, it has been proposed to exploit this metabolic characteristic as a new vector control strategy using inhibitors of tyrosine catabolism [61,63]. Moreover, proteins involved in the metabolism and transport of both arginine and proline (T1HIQ9), glutamine (T1HFR5 and T1HFS7), and lysine (T1HVD2), are overexpressed in the AM after feeding with fold changes ranging from 3 to 7 (Table S1). The increased expression of proteins involved in the metabolism of amino acids after feeding reinforce our suggestion that protein digestion is already initiated in the AM [20]. In the PM, a D-3-phosphoglycerate dehydrogenase (R4FM92), the first enzyme in the L-serine biosynthetic pathway, showed a two-folds increase after blood feeding (Table S1).

- Lipocalins

The lipocalins family is a large group of small extracellular proteins exhibiting great structural and functional variation, both within and between species [64]. In hematophagous arthropods, lipocalins are known for their multifaceted role, especially in saliva. Indeed, they are involved in the inhibition of the platelet aggregation [65], the binding of nitric oxide (NO), and the histamine released by immune cells [66]. Several proteins belonging to this family showed an important induction after blood ingestion in both the AM and PM tissues (Figure 2 and Figure 3). Six blood regulated lipocalins (Figure 2 and Table S1), represented by lipophorin (T1HDK5), triabins (T1H9A1 and T1H7Q9), and nitrophorin (R4G3G5) have been identified in the AM (Table S1). Interestingly, the lipocalin (R4G3A3) with the highest level of induction (15-folds) in this work also corresponds to the lipocalin (Rp-772) with the highest number of transcripts in the AM [40]. Nine blood regulated lipocalins have been identified in the PM (Figure 2 and Table S1), among which eight nitrophorins and a salivary platelet aggregation inhibitor 1 (T1HDI2). Nitrophorins (NPs) are NO carriers reported to play a role in the innate immunity of insects [67]. There are seven different NPs named 1–7, and the most studied ones are NPs 1–4, containing a heme group, which is capable of binding NO [67]. NPs are also involved in other biological activities such as vasodilation, platelet aggregation inhibition, histamine binding, and anticoagulation [68]. The NPs identified in this study belong to NP1, NP3, and NP4. Their level of expression increases drastically ranging from 15- to 26-folds (Figure 3 and Table S1). Although the role of salivary NPs has been well studied, their role in the PM remains poorly investigated. However, their expression in this tissue in response to blood ingestion has been correlated to immune response modulation [69]. In that respect, nitrophorin expression has been induced by *T. cruzi* infection of *T. infestans* midgut [43]. Since the expression of NPs is regulated by the blood meal independently of *T. cruzi*, we suggest that the parasite takes advantage of NPs outcome to develop in the midgut. Despite the large number of NPs identified in the PM and in spite of their high level of induction after blood feeding, none of them were identified in the transcriptomic study [40]. Given the major role of NPs in the physiology of the insect and most probably for the development of *T. cruzi* inside the vector, they could constitute promising targets for anti-triatomine control and parasite development and transmission.

- Proteins involved in transcription and translation machinery

Ten differentially expressed proteins identified in the AM were associated with gene expression processes, nine of them were up-regulated while only a single protein was down-regulated (Figure 2). Among the up-regulated proteins we have identified three subunits forming the multiprotein complex eukaryotic translation initiation factor 3 (eIF3), which is the largest and most complex initiation factor. All subunits forming this complex are essential for translation. In fact, this multiprotein complex stimulates nearly all steps of translation initiation by binding the small ribosomal subunit (40S) [70]. The increase in the expression level of certain proteins involved in gene expression confirms that an immediate response is implemented in response to the arrival of blood, allowing the expression of blood processing machinery. 

#### 4.2.2. A Timid Stress Response Is Induced in the Midgut of *R. prolixus* in the Early Hours Post Blood Feeding

- Oxidative and heat stress response proteins

Blood processing in the digestive tract of hematophagous arthropods induces several stresses such as oxidative stress related to high concentrations of heme released after hemolysis leading to the formation of high amounts of ROS, which can damage biological molecules [71]. *R. prolixus* has a unique and complex heme degradation pathway, which involves a number of enzymes where heme is chemically modified before oxidative cleavage of the porphyrin ring by heme oxygenase [72]. In that respect, the analysis of the proteome of the digestive tract of *R. prolixus* showed the constitutive expression in both the AM and the PM of a very important antioxidant enzymatic machinery in terms of classes of detoxifying enzymes and the number of isoforms expressed in each class [20]. However, only a dehydrogenase (R4G4V3) and an oxidoreductase (T1HF19) were moderately up-regulated (two-folds) in the AM after a blood meal (Table S1) while the expression of a heme-binding protein (Q8T5U0) was more importantly induced (six-folds) (Table S1). The preventive antioxidant role of this protein has been demonstrated in *R. prolixus* [73] as well as its role as a heme transporter [71,74,75]. In addition, silencing of *Rhodnius* heme-binding protein expression had several physiological consequences on the insect such as complete inhibition of molting and impaired embryogenesis [76,77]. Transferrin (B8LJ43), an iron binding transport protein, which plays a crucial role in iron metabolism in both vertebrates and invertebrates [78], is also strongly up-regulated (eight-folds) in the AM after a blood meal (Table S1 and Figure 3). Similarly, transferrin has been shown to be up-regulated in *A. aegypti* cells treated with heat-killed bacterial cells [79], suggesting its potential involvement in the innate immune response of the insect. Interestingly, NADPH-cytochrome P450 reductase (T1HNV5) and a glutaredoxin (T1I2X5) were both down-regulated in the AM following blood meal ingestion with eight- and two-folds, respectively (Table S1). In the PM, the putative sulfotransferase (R4FLL8) and two isoforms of the putative multicopper oxidase (R4FLW6 and T1HE55) had their expression increased by three and five-folds, respectively (Table S1) while a glutathione S-transferase (R4G3S8) and a dihydrolipoyl dehydrogenase (R4G4V3) were only slightly down-regulated (2-folds, Table S1). Whereas cytochrome P450 reductase generates ROS via oxidation of NADPH, glutaredoxins play an important role in maintaining intracellular thiol-redox homeostasis by scavenging ROS. The down-regulation of protein expression level of redox enzymes is compatible with the early decrease of ROS levels observed in the midgut (AM and PM) of *R. prolixus* immediately after a blood meal (1 h). This mechanism was proposed to take place to avoid an oxidative stress induced by heme following a blood meal [80].

In addition to the oxidative stress, the massive ingestion of warm blood causes a drastic increase in body temperature, for which the consequences are diverse [81]. The first molecular response to heat stress is to increase the expression of heat shock proteins (HSPs) [82], which act as chaperonins that ensure the functional folding of nascent proteins [83]. Accordingly, the expression of hsp70 (T1HJT8) and a putative hsp90 (R4FMH8) increased between two to three times after blood ingestion in both AM and PM tissues (Table S1). Several HSPs were found to be overexpressed in the midgut of several hematophagous species after a blood meal such as *A. aegypti* [84], *R. microplus* [85], and *O. erraticus* [36]. Among the HSPs, hsp70 is the most studied in response to environmental heat stress [81,82]. Its knockdown in *R. prolixus* resulted in a radical alteration in the physiological response to a blood meal leading to insect death due to a complete alteration in the blood digestion process coupled to a reduction in energy metabolism and immune response [86]. This suggests that HSPs are necessary not only in heat stress response but also for triggering blood digestion. Exploiting the importance of these proteins in the context of vector control could be envisaged.

- Innate Immunity related proteins

Insects produce a variety of antimicrobial peptides (AMPs) such as defensins, attacins, cecropins, prolixicins, and enzymes (as lysozymes), to combat pathogens. Production of these antimicrobial molecules takes place mainly in the fat body, the hemocytes, and the digestive tract [87,88]. Several c-type lysozymes have been characterized in triatomines with multiple roles such as the digestion of bacteria to regulate the gut flora and the immune response in the hemolymph to prevent pathogen colonization or both [89,90]. Hence, Lys1 (Q2TPW4) from *T. brasiliensis* AM showed a gradual increase in its transcript level post blood feeding to reach a maximum, five days after feeding [91]. Comparably, lys1 (Q7YZS5) from *T. infestans* AM, a homolog of *T. brasiliensis* Lys1, showed the same transcript expression pattern but reaching its maximum 15 days after feeding [89]. Two lysozymes, RpLys-A (A9LN31 = T1IGM2) and RpLys-B (A9LN32), were also characterized in *R. prolixus* [90] showing different tissues and time points expression. RpLys-A which expression level increases substantially in the digestive tract upon bacterial infection in the hemocoel was shown to cluster with the closely related triatomines with digestive lysozymes [90]. On the other hand, RpLys-B, whose expression level increased drastically in the fat body upon bacterial infection in the hemocoel, best aligns with a phylogenic group containing immune-related lysozymes [90]. Surprisingly, the two lysozymes expression level was not affected by a blood meal while a significant increase in RpLys-A expression in the digestive tract was observed when alimented with *T. cruzi* containing blood [90]. In this context, we have identified two lysozymes in the AM (T1IGM2 and A9LN32) which expression increases by four- and five-folds, respectively, post blood feeding. Although our results are in agreement with the transcriptomic data showing their overexpression in the AM, the PM, and the rectum of *R. prolixus* [40], they do not agree with Ursic-Bedoya et al. [90] who showed an induction only upon either bacterial or *T. cruzi* infection. Interestingly, A9LN32 was also identified in the PM proteome of *R. prolixus* [20]; however, its expression level remained unchanged 6 h post-feeding. These observations corroborate with the expression of Lys1 (Q2TPW4) of *T. brasiliensis*, which showed a drastic increase of its transcript expression in the AM while its transcripts were very low in the PM [91]. 

A protein containing an MD-2-related lipid-recognition (ML) domain (T1HU92) related to the recognition of pathogen-related molecules [92] showed a five-fold increase in response to blood feeding. Transcripts containing this domain have been triggered by blood in the midgut of *I. ricinus* [93] and induced in *I. ricinus* whole body after *Borrelia burgdorferi* infection [35]. At the protein level, one ML domain protein was identified in the hemolymph proteome of *A. gambiae* [94]. Therefore, it would be interesting to investigate the role of this protein in the recognition of *T. cruzi*. 

It is noteworthy to mention that although proteins associated with IMD, Toll, and Jak/STAT pathways involved in innate immunity have been identified in *R. prolixus* digestive tract proteomes [20,95,96], the early events post blood feeding resume in the induction of these very few immune-related proteins. Although lysozymes have been suggested to have a role in immunity, the observed up-regulation in the early hours post blood ingestion supports their digestive function. 

### 4.3. New Insights about Unknown Proteins

One third of the blood induced proteins in the AM were classified as proteins with unknown function and present up to 23-fold changes (Figure 2 and Table S1). The characterization of their function, as well as their implication in blood digestion, could provide valuable information on the physiology of the midgut of *R. prolixus* and might help shed light on certain unclear concepts. 

## 5. Conclusions

In this study we report for the first time the early effect of blood meal ingestion on protein expression in the midgut of Chagas disease vector *R. prolixus.* Since the midgut is the primary interface for vector–parasite interaction, this tissue is qualified as a particularly promising target for the development of new triatomine control strategies. To this end, the post-prandial regulation of protein expression in the midgut tissues was investigated by comparative label-free high throughput proteomic approach. Our results show that the ingestion of blood triggers in the first few hours after feeding, the expression of a range of protein families mainly involved in the digestive process. Interestingly, the dynamics of protein expression 6 h after feeding is mostly modulated in the AM than in the PM. This confirms the important role of the AM and its implication in the blood meal processing. However, the pattern of expression of the proteins involved in the process of protein digestion is a time-dynamic process occurring in both AM and PM compartments. Hence, this dynamic could take other dimensions in these two organs along the digestion kinetics. The highlighted proteins, particularly those whose expression is strongly modulated by blood feeding, deserve special attention regarding their function and the biological processes in which they are involved. Indeed, these proteins could be promising targets for an anti-triatomine control strategy. Therefore, functional experiments such as those based on reverse genetics approaches will be necessary to identify the most relevant candidates identified in our proteomics study to enable new ways to interfere with the development of triatomes and the transmission of *T. cruzi.*

## Data Availability

MS raw data as well as the files generated by MaxQuant and Perseus have been deposited to ProteomeXchange Consortium *via* MassIVE repository with the following dataset identifiers (PXD019150) (MSV000085406).

## Supplementary Materials

The following are available online at https://www.mdpi.com/article/10.3390/microorganisms9040804/s1, Table S1: Classification of blood ingestion regulated proteins, identified by differential proteomics analysis in the midgut tissues of *Rhodnius prolixus.*
